# Titratable residues that drive RND efflux: Insights from molecular simulations

**DOI:** 10.1017/qrd.2024.6

**Published:** 2024-04-01

**Authors:** Robert Clark, Kahlan E. Newman, Syma Khalid

**Affiliations:** 1 Department of Biochemistry, University of Oxford, Oxford, UK; 2 School of Chemistry, University of Southampton, Southampton, UK

**Keywords:** antimicrobial resistance, conformational cycling, efflux, molecular dynamics, protonation states

## Abstract

The resistance–nodulation–division efflux machinery confers antimicrobial resistance to Gram-negative bacteria by actively pumping antibiotics out of the cell. The protein complex is powered by proton motive force; however, the proton transfer mechanism itself and indeed even its stoichiometry is still unclear. Here we review computational studies from the last decade that focus on elucidating the number of protons transferred per conformational cycle of the pump. Given the difficulties in studying proton movement using even state-of-the-art structural biology methods, the contributions from computational studies have been invaluable from a mechanistic perspective.

## Introduction

**Figure 1.**
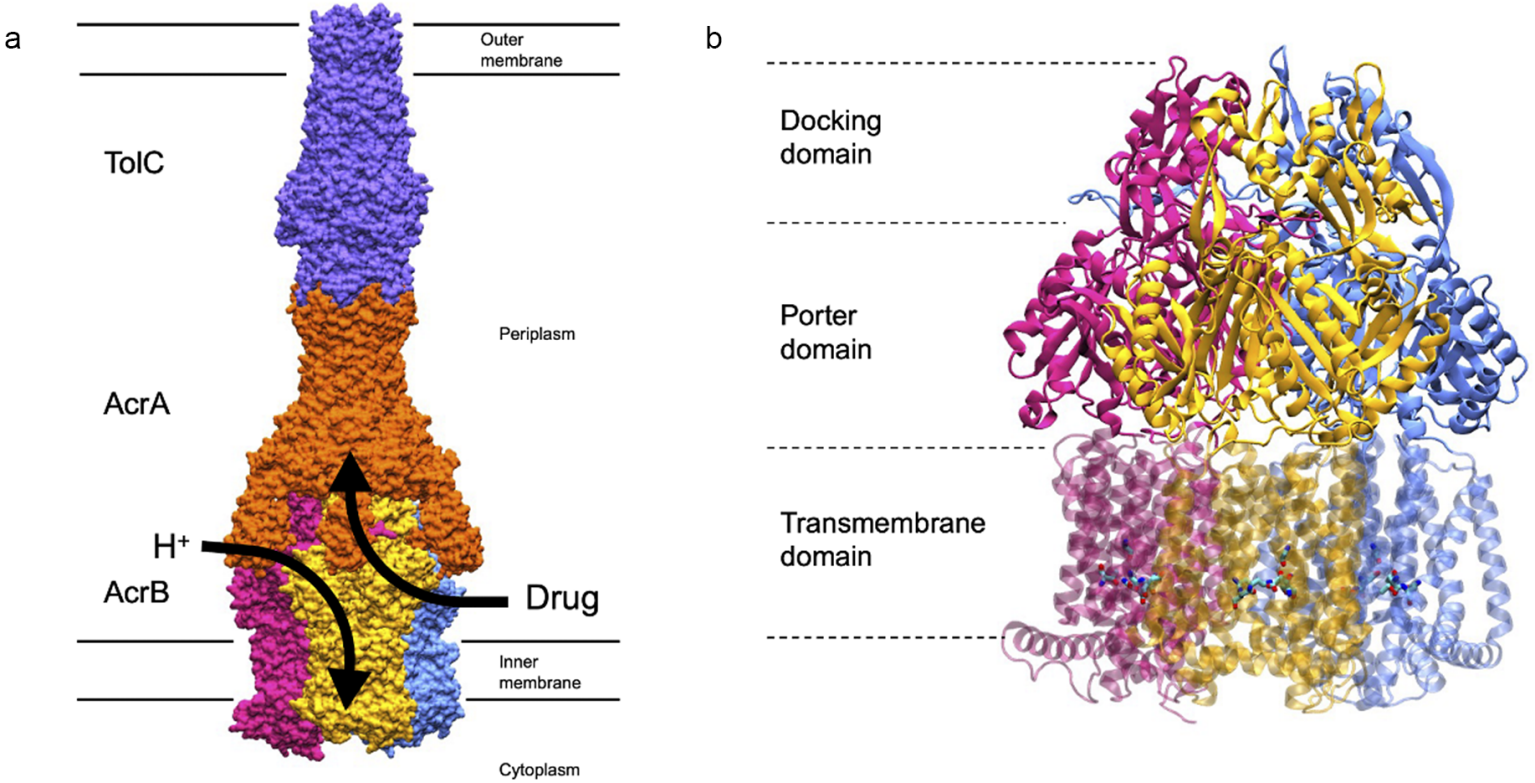
AcrB forms part of the tripartite multidrug efflux pump, AcrAB-TolC. (a) Surface representation of AcrAB-TolC (PDB ID: 5O66; Du *et al.,* 2014) and its situation within the cell envelope of Gram-negative bacteria. AcrB sits within the inner membrane; TolC sits within the outer membrane; and AcrA is the periplasmic adaptor protein. Substrates, such as certain classes of antibiotics, are thought to enter AcrB either in the periplasm near the inner membrane, or from the outer leaflet of the inner membrane directly. Protons enter AcrB from a different entry point, and transit through the TMD to the cytoplasm. The precise mechanism of the proton relay is unclear. TolC is coloured in purple, AcrA is coloured in orange, AcrB is coloured according to state. The Access state is coloured pink, the Binding state is coloured yellow, and the Extrusion state is coloured in blue. (b) Cartoon representation of AcrB (PDB ID: 4DX5; Eicher *et al*., 2012). AcrB has three main domains: the Docking domain which interacts with AcrA; the Porter domain contains the drug entry site; and the Transmembrane domain uses the proton motive force to induce drug extrusion. The titratable residues thought to orchestrate proton transfer are shown in stick representation and coloured according to element.

Gram-negative bacteria, which comprise two-thirds of ESKAPE pathogens, enhance their antimicrobial resistance profiles using resistance–nodulation–division (RND) efflux machinery. These tripartite protein complexes span the cell envelope and confer multidrug resistance by expelling molecules from within the periplasm/proximal to the inner membrane into the extracellular milieu, thereby reducing intracellular accumulation of antibiotics (Figure 1*a*). Such machinery has thus been the focus of intense research in an attempt to better understand the process of efflux (Alav *et al*., 2021). The most widely studied of these systems is AcrAB-TolC from the archetypal Gram-negative bacterium, *E. coli.* AcrB, the homotrimeric RND protein of this assembly, drives substrate recognition and extrusion (Figure 1*b*). AcrB protomers cycle the Access/Loose, Binding/Tight, and Extrusion/Open states to bind substrates and expel them into the periplasmic adaptor protein (AcrA) before diffusing out of the cell via the outer membrane factor (TolC). In this review we will refer to these RND protomer states only as Access (A), Binding (B), and Extrusion (E).

**Figure 2.**
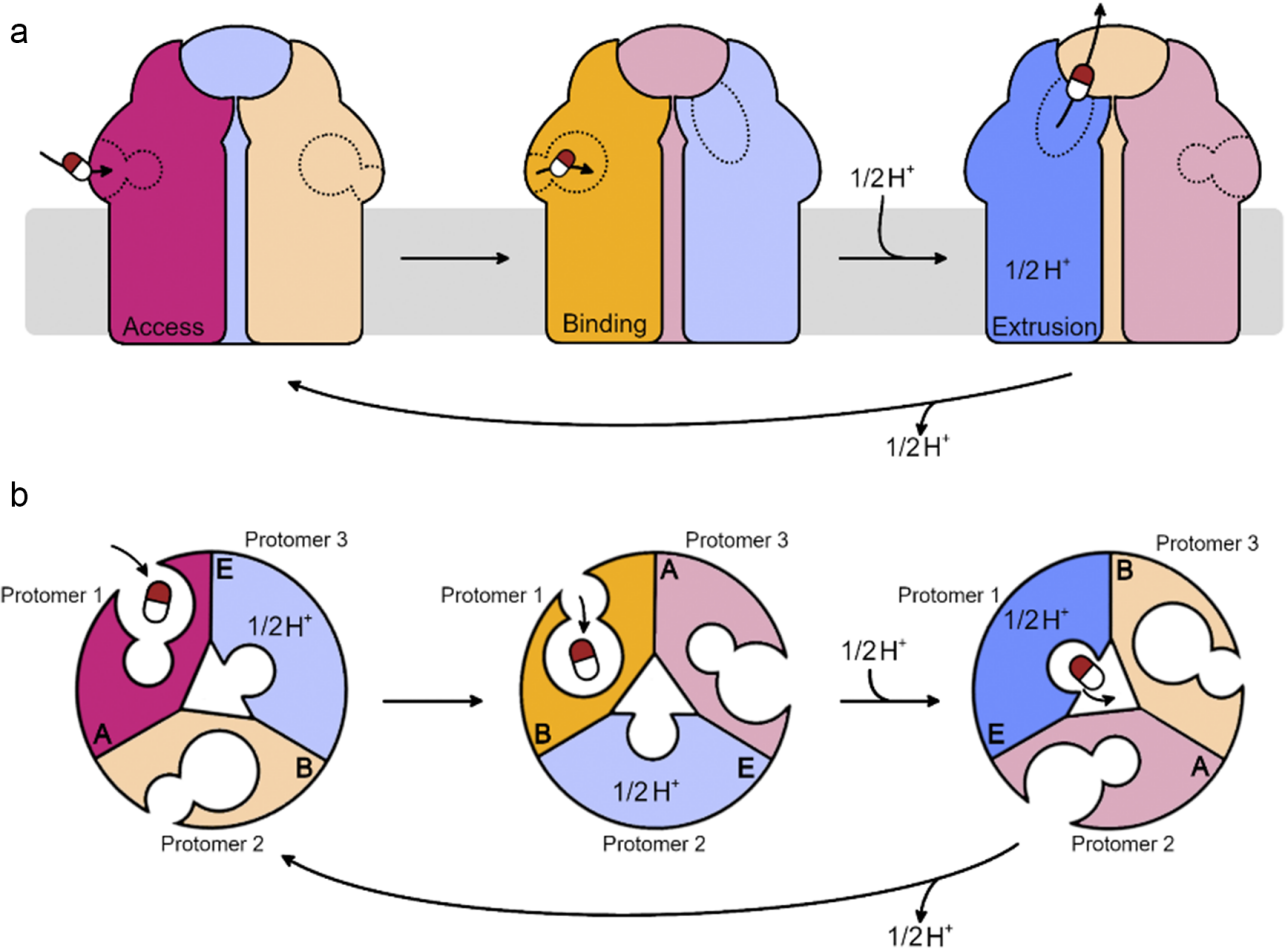
Cartoon schematic of functional rotation and substrate transport through inner membrane RND transporter proteins. (a) Side-view of the RND protein in the inner membrane. (b) Top-down (from the periplasm) view of the porter domain. For visual clarity, protonation and drug extrusion are considered for a single protomer only. Substrates enter the proximal binding pocket of the Access protomer from the periplasm/periplasmic leaflet of the inner membrane. Substrate binding induces a conformational change in the protomer to the Binding state, and the substrate moves to the distal binding pocket. Protonation then occurs in the relay in the transmembrane domain, inducing a conformational change to the Extrusion state. The periplasmic cleft closes and the exit gate opens, allowing the substrate to exit into the periplasmic adaptor protein (not shown).

**Figure 3.**
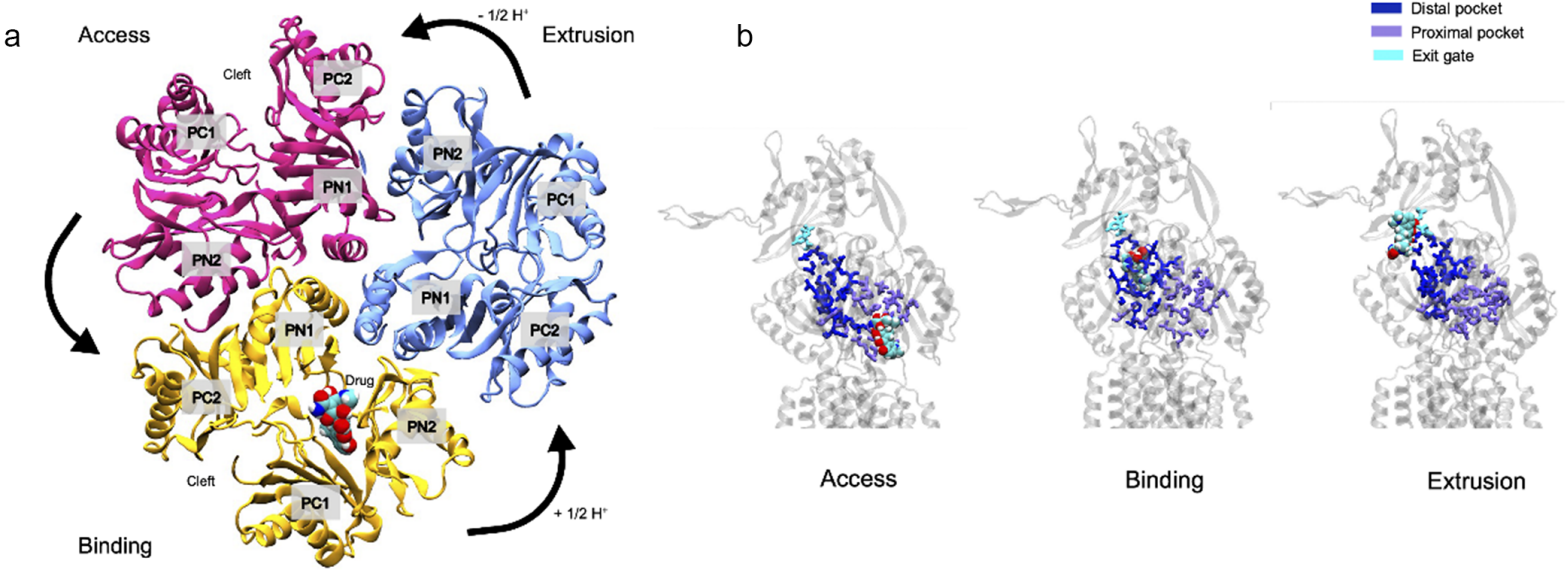
AcrB undergoes a conformational cycle that functionally connects the movement of protons through the protein to drug extrusion. (a) Porter domains of AcrB viewed from the periplasm, showing the different conformational changes that correspond to drug access, binding and extrusion. The Access state is coloured pink, the Binding state is coloured yellow, and the Extrusion state is coloured in blue. Adapted from Matsunaga *et al.* (2018). (b) Drugs are thought to move through the proximal binding pocket (purple) in the Access state, bind to the distal pocket (dark blue) in the Binding state, and leave through the exit gate (cyan) in the Extrusion state. A drug (minocycline), is shown moving through these different regions. The drug was placed manually in the Access and Extrusion states for illustrative purposes, but the position of the drug in the binding state comes from PDB ID:4DX5 (Eicher *et al*., 2012). The definition of the different regions comes from Vargiu and Nikaido (2012).

During conformational cycling, the periplasmic cleft, defined by the porter subdomains PC1 and PC2, is open in the Access and Binding states (Figures 2 and 3*a*), while the exit gate in the docking domain is closed. It is thought that drugs are bound in a proximal binding pocket in the Access state before moving to the distal pocket to induce transition of the protomer to the Binding state (Figures 2 and 3*b*). Transformation to the Extrusion state results in the closing of the periplasmic cleft and the opening of the exit gate through which the drug is extruded into the periplasmic adaptor protein. The B to E transition is putatively an energy-dependent step (Seeger *et al.,* 2008), powered by proton motive force: protons are transferred from the periplasm to the cytoplasm *via* the transmembrane domain (TMD). Buried within the TMD are essential/highly conserved polar/charged residues that constitute a proton relay network, facilitating proton transfer across this otherwise hydrophobic region. D407, D408, K940, R971, and T973 have been identified as essential to function (Murakami and Yamaguchi, 2003; Takatsuka and Nikaido, 2006), with nearby N941, G403, and S481 also highly conserved. The exact mechanism of proton transfer, proton stoichiometry for each full cycle, and which of the acidic residues is protonated at each stage of the cycle remains uncertain.

## Structural data and early simulation studies

Computational studies have been widely used to investigate efflux machinery (Athar *et al.,* 2023). Early simulations did not focus explicitly on the proton relay network, instead utilising postulated protonation states (Seeger *et al.,* 2008). Simulations by Fischer and Kandt (2011) demonstrated alternating access for water (important for mediating proton transfer) entering the TMD from the periplasm and cytoplasm in different protomer states. Further simulations by the same group displayed flexibility in the porter domain, with opening and closing of the periplasmic cleft (Fischer and Kandt, 2013). For an extensive discussion of RND simulation studies, we direct the reader to a recent review (Athar *et al.,* 2023). Here we turn our attention only to simulation studies where the protonation states of the proton relay network residues have been a primary focus.

**Figure 4.**
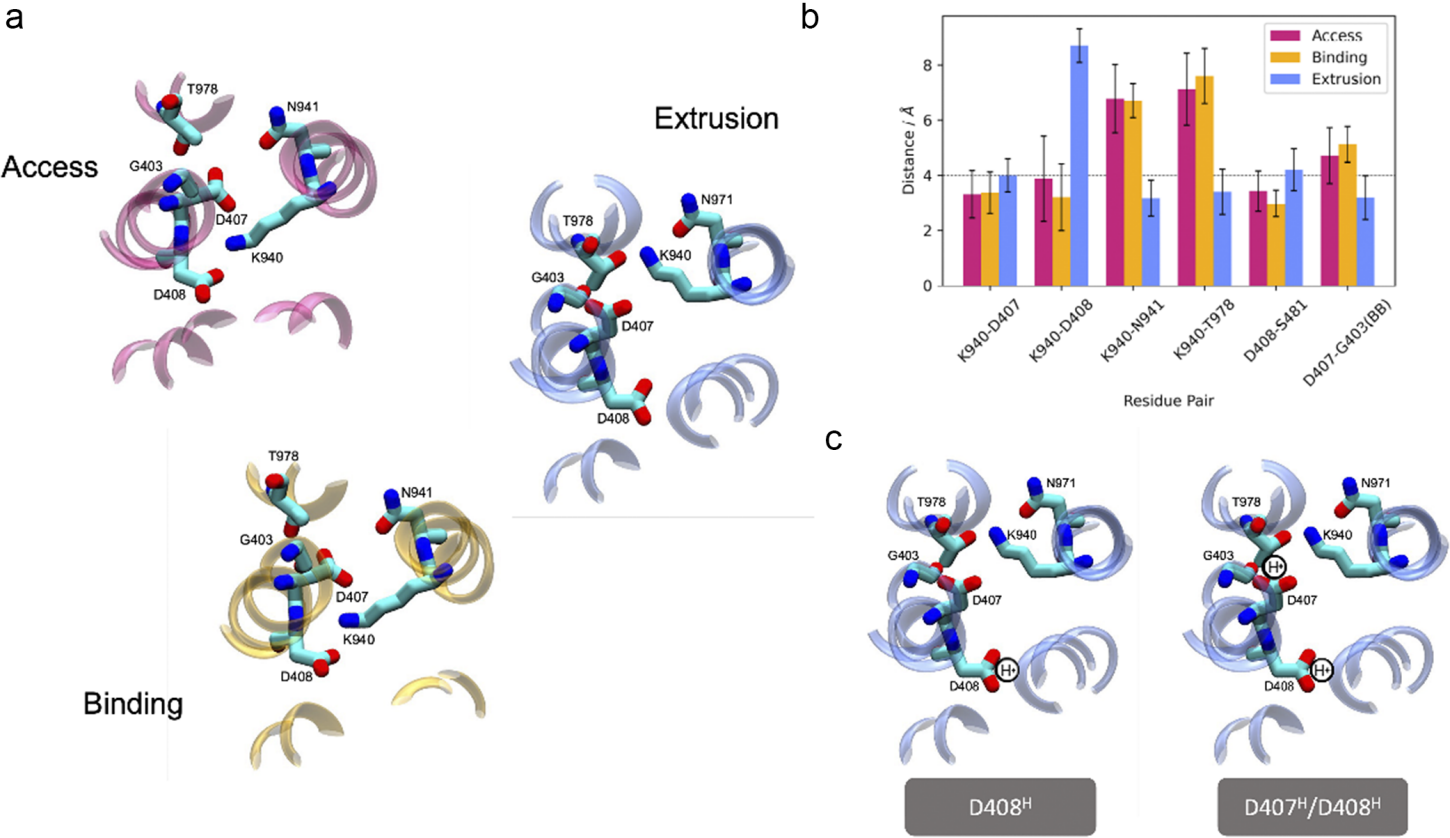
The titratable residues of AcrB adopt different configurations in different states. (a) Close-up view of key residues of the proton relay. Each image is rotated, so viewed from the same orientation. In the Access and Binding states the relay network is considered ‘engaged’, and in the Extrusion states the network is considered ‘disengaged’. Adapted from Matsunaga *et al.* (2018). (b) Inter-residue distances for residues in the proton relay network of Gram-negative bacterial RND protein structures deposited in the PDB (raw data and relevant references available in the Supplementary material). Distances were calculated as the shortest distance between side chain oxygen/nitrogen atoms, except for G403 where the backbone carbonyl oxygen was used. (c) Conformational transition from the Binding to the Extrusion state is proposed to proceed *via* one protonation event (D407^−^/D408^H^) or two (D407^H^/D408^H^). Structures shown in (a) and (c) are of PDB ID: 4DX5.

As there are two titratable residues in the relay network (D407 and D408), it is assumed that the number of protons transferred will be one or two (Alav *et al*., 2021). Based on structural data, the current consensus is that both D407 and D408 are deprotonated in the A and B protomers: K940 sits between these two residues, within salt-bridging distance of both (Figure 4*a,b*). This relay network arrangement has been denoted the ‘engaged’ state. In the Extrusion protomer, K940 orients away from the aspartates and towards T978/N941 (‘disengaged’ state, Figure 4*c*). The protonation states of D407 and D408 in the Extrusion protomer remain unclear and cannot be elucidated explicitly *via* state-of-the-art experimental structure-determination techniques as hydrogens are not well-resolved. Protonation may be inferred from side-chain orientations, but this can yield ambiguous or conflicting results. In the Extrusion state the distance between the G403 backbone oxygen and D407 carboxylate (<4 Å, Figure 4*b*) suggests D407 is protonated to participate in hydrogen bonding. Simultaneously, the D407 carboxylate is also within salt-bridging distance (~4 Å) of the K940 amine nitrogen, implying D407 is deprotonated! Experiments such as carbodiimide labelling to test protonation states (Seeger *et al.,* 2009) can be a powerful investigative tool, but the bulky reagents are restricted in the narrow water wires of the TMD. Modelling and molecular dynamics (MD) simulations have been used to address this knowledge gap. These studies generally fall into two categories, those that support: single proton transfer; or transfer of two protons per conformational cycle (Figure 4*c*).

## Two protons

Computational support for protonation of both D407 and D408 is based largely on structures and simulations presented by Pos and colleagues (Eicher *et al*., 2014). Eicher *et al.* extended the analysis of their previous crystal structure (4DX5 (Eicher *et al*., 2012)) to focus on different conformations adopted by key residues of the AcrB TMD, including D407 and D408. They compared the different structures and calculated likely protonation states of key residues using two separate approaches.

Ten independent Metropolis Monte Carlo simulations (Brooks *et al.*, 2009) were conducted to evaluate protonation states of K940, R971, and all aspartate, histidine and glutamate residues. Energies were evaluated using the Poisson-Boltzmann equation. Each simulation comprised five cycles: 100,000 steps for annealing, starting at 400 K to encourage greater exploration, followed by 500,000 steps at 300 K for equilibration. Proposed changes in protonation states were accepted or rejected based on the Metropolis criterion. By averaging the protonation state over the final 500,000 steps across all 10 simulations, a global average was obtained. The periplasmic pH was assumed to be 5.5 and the cytoplasmic pH 7.5, with a dielectric constant (εp) of 4. It was noted a pH of 7.5 across the protein led to analogous results. These simulations predicted that in the E state, D408 would be protonated 100% of the time and D407 90% of the time. The study also evaluated the likelihood of protonation for the mutants K940A, R971A, D407N and D408N. When present, both D407 and D408 were predicted to be protonated in the E states. These mutants were shown previously to be functionally inactive (Guan and Nakae, 2001; Takatsuka and Nikaido, 2006): Pos and colleagues related the change in activity of these mutants to alterations in the proton relay network.

Acknowledging the dependence of this electrostatics approach on dielectric constants, free energy perturbation (FEP) was also used to re-evaluate the protonation states of key residues. All-atom simulations of the relevant AcrB protomers were used, alchemically transforming the protonation state of specific residues to calculate the change in potential energy of the system. pK_a_s were calculated using known side chain analogues, yielding values of 8.2 and 10.8 in the E state for D407 and D408, respectively. This agreed with their protonation probability prediction that both D407 and D408 were protonated in the E state, and matched crystal structure side chain placements (Figure 4*a*). It should be noted there were large errors for D407, indicating that the calculations may not be fully converged (Table 1): while FEP can be accurate, it can also be limited by sampling issues which would bias results towards the simulation starting states (Coskun *et al*., 2022).

**Table 1.** Varied pKa values were calculated for D407, D408 and K940 in the studies presented in the text

Paper	Yamane *et al*. (2013)				Eicher *et al*. (2014)	Yue *et al.* (2017)	This work	
Method	PROPKA v2.0	PROPKA v3.0	MCCE; ε*p = 4*	MCCE; ε*p = 8*	FEP	CpHMD	PROPKA v3.5	PROPKA v3.5
PDB ID	2DHH	2DHH	2DHH	2DHH	4DX5	4DX5	2DHH	4DX5
	pKa							
D407 (A)	4.52	3.88	<0	<0	N/A	2.4	3.88	3.41
D407 (B)	5.24	4.07	<0	<0	N/A	2.5	4.07	3.46
D407 (E)	4.91	6.57	0.76 ± 0.07	1.20 ± 0.05	8.2 ± 2.1	3.5	6.71	7.86
D408 (A)	3.32	5.41	6.95 ± 0.18	4.89 ± 0.12	N/A	2.8	5.41	4.65
D408 (B)	3.28	5.89	6.32 ± 0.32	5.01 ± 0.07	N/A	2.7	5.89	4.68
D408 (E)	7.1	8.77	9.88 ± 0.12	7.06 ± 0.07	10.8 ± 0.45	4.3	8.75	9.14
K904 (A)	11.44	12.2	>14	>14	N/A	N/A	12.2	12.35
K904 (B)	11.26	11.95	>14	>14	N/A	N/A	11.95	12.13
K904 (E)	8.51	8.89	>14	>14	8.8 ± 0.38	N/A	8.89	8.17

Methodology and choice of starting structure can lead to varied results. The highlighted residues are those of the key E state. Also included are values calculated for the AcrB structures 2DHH and 4DX5 using PROPKA v3.5 (Olsson *et al*., 2011; Søndergaard *et al.,* 2011).

Eicher *et al.* confirmed two protonation events would create an alternating access water wire, facilitating proton transfer to and from solution. Using unbiased all-atom MD, membrane-bound AcrB was simulated using the predicted protonation states. Access to the periplasmic and cytoplasmic solvent was found to alternate with conformational state (Fischer and Kandt, 2011). Overall, the evidence presented in the study is consistent with a two-proton model.

The two-proton model has also been tested on a homologue of AcrB. A recent paper by Fairweather *et al.* investigated substrate binding and the effect of protonation on the RND protein MtrD (*Neisseria gonorrhoeae*, 4MT1; Bolla *et al*., 2014; Fairweather *et al*., 2021). Studying the role of protonation, united atom (UA; Schmid *et al*., 2011) conventional MD simulations were used, changing the protonation states of D405 and D406 (AcrB D407/D408 analogues) and the presence of progesterone substrate molecules. In the UA representation, five systems were simulated with varying protonation states and number of substrates. In the absence of substrates and protonation, MtrD adopted a symmetric A/A/A state – the putative resting state (Su *et al.,* 2006). Protonation of both aspartates in the absence of a substrate was sufficient to begin the opening of the exit gate (distance distributions with peaks at ~8.6 and ~ 12 Å, where the closed gate is ~5–6 Å) in the same monomer but caused instability at the relay site (K948-T985 distance distribution displays three peaks) and did not induce conformational cycling in neighbouring protomers.

Substrate binding accelerated transitions towards the asymmetric structure. Protonation in the extrusion protomer and binding of a single progesterone in the proximal binding pocket of an adjacent protomer stabilised an asymmetric state (reduced fluctuations in K948-T985 and exit site distances) but did not allow complete transition on the sampled timescales. The presence of progesterone in the binding pockets of both adjacent protomers allowed further asymmetry to develop: binding of substrates in two protomers, and protonation of the final protomer was required for the functional rotation of MtrD to be observed. Binding of multiple substrates has been shown to accelerate conformational cycling in other simulation studies (Wang *et al.,* 2015; Matsunaga *et al.,* 2018). Consistent with studies on AcrB, protonation of both aspartates induced rotation within the TMD, and the most extensive water pathways were observed in the binding protomer (Fischer and Kandt, 2011; Eicher *et al*., 2014). While this study yielded results suggesting protonation of both residues (and the presence of >1 substrate) can induce conformational cycling, the authors noted the proton relay network was never fully disengaged, despite protonation of D405 and D406. Two explanations were offered: the timescale of this transition may be beyond that sampled; or disengagement of the relay network may be driven by a different proton stoichiometry, that is, the two-proton model may not be appropriate for MtrD.

## One proton

In 2013, Ikeguchi and colleagues used conventional all-atom MD simulations to investigate the possible protonation states of the titratable residues of AcrB (2DHH; (Murakami *et al.,* 2006; Yamane *et al.,* 2013). All possible combinations of protonation states for D407/D408 were tested in the E state protomer of this asymmetric structure. While each simulation was only 100 ns, they were able to observe differences in stability of the E-state depending on the protonation pattern. Their results suggested only D408^H^ D407^−^ stabilised the E-state with a disengaged relay network, as determined through inter-residue distances defined by K940, D407, D408, and T978. Protonation of only D407 led to immediate rearrangement of K940: the K940-T978 hydrogen bond was disrupted, and a salt bridge was formed between K940 and E408 which became unstable after 50 ns. D408^H^ D407^H^ was also classified as unstable as K940 became dynamic, switching interactions between D408 and T978 frequently, in addition to the salt bridge between K940 and E407 becoming unstable after 90 ns. The rearrangements and subsequent instability in both systems implies that protonation of D407 is incompatible with the E-state of this crystal structure.

They further probed these findings with two D408^−^ D407^−^ simulations from snapshots taken from the D407^−^ D408^H^ simulation. Deprotonation resulted in immediate re-engagement of the relay network, consistent with a transition to the A state. Principal component analysis of porter domain dynamics supported these results: D407^−^D408^H^ remained stable in the E state, whereas D407^−^ D408^−^ displayed a structural transition from E towards A. Together these results suggested that protonation of D408 alone stabilised the E state, and its deprotonation was sufficient to induce the transition to the A state. As stated by the authors, one weakness of their approach was the limited simulation time; the MD was unbiased, and consequently the timescale sampled was insufficient to explore full conformational transitions. We note that while all possible combinations of states were simulated, only one replicate of each system was reported.

In support of their conclusions, pK_a_ values for the two aspartates were calculated using PROPKA (Li *et al.,* 2005) and multiconformation continuum electrostatics (MCCE; Song *et al.,* 2009). Both methods predicted D408 to be more readily protonated than D407, and at pH 7 only D408 would be protonated. There was a disparity in the values predicted, dependent on the method and selected parameters (Table 1). The difference in values highlights the large effect dielectric constants can have on resultant pK_a_s (Chan *et al.,* 2012).

Jewel *et al.* (2017) simulated the conformational changes of apo-AcrB utilising a hybrid coarse-grain regime. AcrB (asymmetric, 2DHH (Murakami *et al.,* 2006) was modelled in the UA PACE forcefield (Han *et al.,* 2010), and surrounding lipids and water molecules were represented in the MARTINI 2 forcefield (Marrink *et al*., 2007). Due to its coarse-grained nature, this regime can sample longer timescales than atomistic simulations, with the caveat of reduced resolution. Similar to the methodology of Yamane *et al.* (2013), this study explored the different combinations of permanent protonation states for D407/D408 in the extrusion protomer: both deprotonated, one protonated, or both protonated, for a total of four systems. When both residues were deprotonated in the E protomer, the relay network approached an engaged (Access) state: K940 moves away from T978 to form salt bridges with D407 and D408 within 100 ns. Extending the simulations to the microsecond timescale, this system also displayed closing of the exit channel (Y758–Q124 decreases from ~12 Å to ~5 Å) and opening of the periplasmic cleft, again consistent with an E → A transition.

Protonation of D408 was found to maintain the orientation of the residues observed in the extrusion state of the crystal structure (K940 was always closer to T978 than D408); the exit channel remained open; and the periplasmic cleft remained closed. This contrasts with systems where D407 was protonated: in both cases, the residues of the relay network were described as *“rather dynamic”*, and the exit channel opens to a greater extent than that observed in the crystal structure (>15 Å vs ~ 12 Å in the crystal structure). It was noted that this may be due to the coarse-graining scheme or the relatively large time step (5 fs), but may also indicate instability of the E-state with D407^H^. Taken together, these results are consistent with the atomistic study by Yamane *et al.* (2013): protonation of D407 destabilises the E state; protonation of D408 stabilises the E state; deprotonation of D408 drives the Extrusion to Access transition.

Following on from this, Jewel *et al.* (2020) used the same hybrid coarse-graining regime to explore conformational changes induced by changes in protonation state and the presence of an indole substrate. Four systems were simulated, except this time protonation states were chosen to induce the B → E transition (rather than E → A). Three protonation schemes for the binding monomer were simulated with indole present in the binding site of that same monomer: D407^−^ D408^−^; D407^−^ D408^H^; D407^H^ D408^−^. The fourth system had all D407/D408 residues deprotonated, and no substrate. Both systems with protonated aspartates displayed closing of the periplasmic cleft (measured by a large increase in the T676-F563 distance) and an opening of the exit channel (Q124 and Y758 distance increase to >12 Å), suggesting a B → E transition. Interestingly, the system with bound indole but no protonation of D407/D408 also displayed opening at the exit channel, though the periplasmic cleft remained open. In the absence of substrates or protonation, the final system tended towards the A/A/A state with a closed exit channel and open periplasmic cleft: the putative resting state of RND proteins (Su *et al.,* 2006). These results suggest that protonation of either D407 or D408 or the binding of a substrate could induce conformational cycling in the efflux pump.

Constant pH MD (CpHMD) has also been used to investigate these residues. Yue *et al.* (2017) used hybrid-solvent continuous CpHMD to investigate the protonation states and conformational dynamics of the TMD of AcrB. Only the TMD of AcrB (4DX5 (Eicher *et al*., 2012) was simulated to reduce computational cost and ensure adequate sampling. The pH replica exchange protocol (Wallace and Shen, 2011) was used, with 24 replicas over the pH range 1–8.5. While conventional MD assumes fixed protonation states which must be selected *a priori*, CpHMD allows titratable residues to be protonated/deprotonated over the course of the simulations according to the solution pH and local environment. CpHMD also enables the calculation of the pK_a_ values of all titratable residues simultaneously, in contrast to traditional FEP methods which yield only one pK_a_ at a time while fixing the protonation states of the other residues. In this case, CpHMD was coupled with replica exchange to achieve convergence of pK_a_ values: by exchanging systems at varying pH values, each utilising different random walks, increased sampling of the potential energy surface of the system is achieved by accelerating the crossing of energy barriers.

Using this method, several observations were made on the pH-dependent behaviour of the titratable residues. Firstly, at pH < 4.0 the native E-state is maintained, but as pH increases a new conformational state (denoted O*/E*) is sampled: at low pH the proton relay network is disengaged (K940-T978 hydrogen bonding), but at higher pH, where both D407 and D408 are deprotonated, K940 adopts an orientation more consistent with the A-state. A large-scale motion accompanies this change: a lateral rotation within the TMD resembling the E → A transition, also observed by Eicher *et al*. (2014).

pK_a_ calculations were consistent with D407^−^/D408^−^ in the A and B states (pK_a_s of 2.2/3.0 in A; 2.6/2.8 in B). Due to their spatial proximity and similar pK_a_s, protonation of these residues is strongly coupled and D407/D408 were considered as a dyad. Titration was therefore described by a stepwise model with two macroscopic pK_a_s: the first/second protonation events in the B state had pK_a_s of 3.4/2.1. Separation of 1.3 units suggests that binding of one proton is more likely than two in the B → E transition. In the E state, the pK_a_s of the two protonation events increase to 5.0/3.4, with D408 having the greater pK_a_ (D407 3.4 vs D408 5.0) and the greater increase in pK_a_ (+0.8 for D407 vs + 2.2 for D408). While the authors note that their pK_a_ values were systematically underestimated, the relative order of the values and the difference between them were considered robust: taken together the results suggest that only D408 accepts a proton in the B → E transition.

Free energy calculation methodologies were used by Matsunaga *et al.* (2018) to investigate the pathway of AcrB functional rotation. They hypothesised that D408 protonation/deprotonation drives the conformational cycling. Two systems were generated, both starting in the BEA state (4DX5 (Eicher *et al*., 2012)) with minocycline bound in the B protomer. These systems were driven towards the EAB state using targeted all-atom MD (Klauda *et al*., 2010; Best *et al.,* 2012). In the first system the D408 of protomer II (E) was protonated, predicted to stabilise the initial BEA state. In the second system, D408 of protomer I (B) was protonated, predicted to induce functional rotation to the EAB state (Figure 3*a*) and hence stabilise the end state. Making use of the string method (Branduardi and Faraldo-Gómez, 2013) and umbrella sampling (Torrie and Valleau, 1977), the minimum free energy pathway between the initial and final states was calculated. It was demonstrated that the local energy minimum for system 1 lay close to the initial BEA state while for system 2 the local minimum was closer to the EAB state, concluding that protonation of D408 of the B state could drive functional rotation to the E state.

To assess how the protonation state affected the free energy, system 1 was alchemically transformed towards system 2 by protonating D408 in protomer I and deprotonating D408 in protomer II. Due to restraints on collective variables (Cartesian coordinates of Cα atoms of the porter domain, TMHs accommodating the relay network, and a flexible loop between TMHs 5 and 6; side chain atoms of D407 and D408), free energy calculations were stated to evaluate the contribution of protonation to any free energy differences. By protonating protomer I (B), the free energy of the minimum was increased by 25.9 ± 0.5 kcal/mol. Electrostatic potential maps showed that the TMD of protomer I was destabilised upon protonation due to repulsion between D408^H^ and proximal cationic residues (K940, R971). The free energy change on transforming the protonation state of system 2 into that of system 1 was also evaluated, but this had a reduced effect (0.4 ± 6.9 kcal/mol), suggesting protonation may no longer affect energetics at this stage in the cycle.

This study further related protonation to conformational changes in the TMD. Comparing the water distribution at the local energy minima of system 1 and system 2 showed the sheer vertical motion had created alternating access to the periplasm and cytoplasm: a possible proton release pathway consistent with previous findings (Fischer and Kandt, 2011). The sheer motion and TM helix tilts were also correlated to porter domain opening/closing using mutual information analysis (McClendon *et al.,* 2009). This study does not discount the possibility that D407 may be protonated during conformational cycling. However, it does demonstrate that singular protonation of D408 in the B state can cause functional rotation of AcrB to a stable protonated E state, and that this functional rotation is translated to the porter domain.

## Conclusions & outlook

Here we have presented a series of computational studies aiming to further elucidate details of the proton transfer mechanism in RND transporters. The literature from the last decade generally favours a one-proton model in which only D408 becomes protonated on transition from the Binding to the Extrusion state, and its deprotonation is sufficient to induce cycling back to the Access state. However, inconsistencies across these studies and conflicting experimental data remain, which cannot currently be reconciled. For example, we compared the pK_a_s estimated by PROPKA v3.5 for 2DHH (Murakami *et al.,* 2006) and 4DX5 (Eicher *et al*., 2012), finding the E state D407 in particular had differing results (6.71 vs 7.86, respectively). Due to different crystallisation conditions and ligands present, it is hard to say whether the structures are within the same native ensemble or perhaps reflect different states (Best *et al.,* 2006). In general, the pK_a_s shown in Table 1 highlight the difficulty in determining protonation states, as each method carries with it its own biases (Coskun *et al*., 2022; Wilson *et al.,* 2023). It was recently demonstrated that all commonly used high throughput pK_a_ estimation approaches, including PROPKA and electrostatics-based methods, are prone to inaccuracies (Wei *et al.,* 2023), so it is not trivial to tease apart these details. Incorporating well-sampled ensemble states with accurate pK_a_s will be essential to accurately assess protonation states. Additionally, some simulation studies have shown substrate binding to accelerate or even be required for full conformational cycling (Wang *et al.,* 2015; Fairweather *et al*., 2021). The interdependence of protonation events and substrate recognition is yet to be fully understood. Finally, it is important to note that simulated systems will inevitably simplify true biological system complexity: studies presented here have simulated the RND transporter isolated in simple symmetric phospholipid bilayers. Of particular importance is the local environment. Not only will AcrB dynamics be affected by coupling to substrates, AcrA, and TolC *in vivo*, but the local proton gradient across the inner membrane will affect the likelihood of protonation for these essential residues. With the maturation of increasingly powerful experimental techniques and simulation studies able to capture greater system complexity, we anticipate further exploration of this family of proteins and their functional mechanism in the future.

## Supporting information

Clark et al. supplementary material Clark et al. supplementary material

## Supplementary material

The supplementary material for this article can be found at http://doi.org/10.1017/qrd.2024.6.
