# Supplementary material for: Titratable residues that drive RND efflux: Insights from molecular simulations
Source: QRB Discov. 2024 Apr 1;5:e5. doi: 10.1017/qrd.2024.6 (PMC11058585; doi:10.1017/qrd.2024.6)
Supplement: Peer review history

# Editor report: Decision: Titratable residues that drive RND efflux: Insights from molecular simulations — R0/PR2

**DOI:** 10.1017/qrd.2024.6.pr2

**Affiliations:** Bioengineering and Chemistry, University of California System, United States

Minor revisions

# Editor report: Decision: Titratable residues that drive RND efflux: Insights from molecular simulations — R1/PR4

**DOI:** 10.1017/qrd.2024.6.pr4

**Affiliations:** Bioengineering and Chemistry, University of California System, United States

No accompanying comment.
